# The expanding epidemic of HIV-1 in the Russian Federation

**DOI:** 10.1371/journal.pmed.1002462

**Published:** 2017-11-28

**Authors:** Chris Beyrer, Andrea L. Wirtz, George O’Hara, Nolwenn Léon, Michel Kazatchkine

**Affiliations:** 1 Center for Public Health and Human Rights, Department of Epidemiology, Johns Hopkins Bloomberg School of Public Health, Baltimore, Maryland, United States of America; 2 Haverford College, Haverford, Pennsylvania, United States of America; 3 Office of the UN Secretary-General’s Special Envoy on HIV/AIDS in Eastern Europe and Central Asia, Geneva, Switzerland; 4 Global Health Center, the Graduate Institute of International and Development Studies, Geneva, Switzerland

## Abstract

In a Perspective, Chris Beyrer and coauthors discuss the threat of HIV to health in the Russian Federation.

## Introduction

In 2017, the Russian Federation (RF) is estimated to have the largest number of HIV-1 infected citizens of any country in Europe [1]. Cumulative reported diagnoses reached over 1.16 million infections by mid-2017, and actual infections, including those that remain undiagnosed and/or unreported, are doubtless substantially higher [2]. In contrast to the global epidemic pattern, the HIV epidemic in the RF and in most countries of Eastern Europe and Central Asia continues to expand significantly. Over 103,000 new HIV diagnoses were reported in the RF in 2016, a 5% increase in new infections over the previous year [2]; reported HIV diagnoses had been increasing at some 10% per year from 2011–2016 [2]. Among Russian men aged 30–39 years of age, a group that has the highest male infection burden, some 2.8% were living with HIV infection in 2016 [2]. AIDS deaths, too, are rising and now negatively impact life expectancy [3]. From January to June 2017, some 14,631 AIDS deaths were recorded, a 13.5% increase over the previous 6-month period [3]. HIV/AIDS has risen to feature in the top 10 causes of premature death in the RF—a 35% increase from 2005 [4]. These realities should concern all who seek global control of the HIV pandemic.

Despite the severity of this epidemic, including its scale, scope, and trajectory, remarkably little attention has been paid to the associated public health crisis in the international scientific literature. This may in part be due to the limited availability of data on HIV-1 in the RF that are presented and published outside Russia, and to the few international collaborations on HIV in the RF under the current administration. Therefore, we reviewed publicly available data in the Russian language on HIV-1 in the RF through mid-2017 and analyzed Russian federal and oblast (province)-level HIV policies and programs to assess the current burden of HIV-1 prevalence and incidence, the state of prevention programs as they relate to the epidemiology of HIV in the country, and policy impacts of current Russian laws, policies, and practices on the future trajectory of the epidemic.

**Fig 1:**
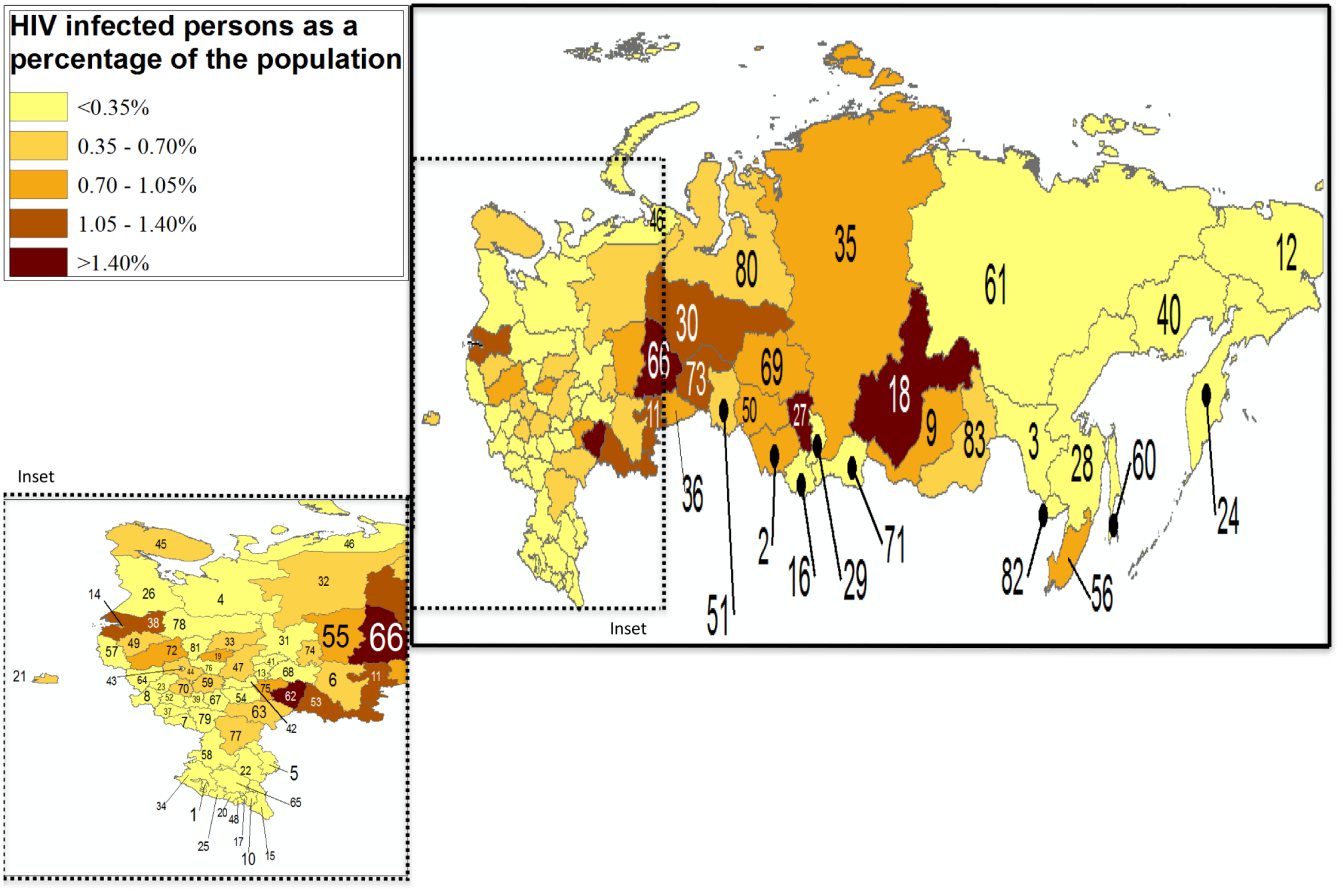
Cumulative HIV diagnoses in the Russian Federation in 2016 [2,6]. Labels: 1—Republic of Adygea, 2—Altai Krai, 3—Amur Oblast, 4—Arkhangelsk Oblast, 5—Astrakhan Oblast, 6—Republic of Bashkortostan, 7—Belgorod Oblast, 8—Bryansk Oblast, 9—Republic of Buryatia, 10—Republic of Chechnya, 11—Chelyabinsk Oblast, 12—Chukotka Autonomous Okrug, 13—Chuvash Republic, 14—City of St. Petersburg, 15—Republic of Dagestan, 16—Altai Republic, 17—Republic of Ingushetia, 18—Irkutsk Oblast, 19—Ivanovo Oblast, 20—Kabardino-Balkar Republic, 21—Kaliningrad Oblast, 22—Republic of Kalmykia, 23—Kaluga Oblast, 24—Kamchatka Krai, 25—Karachay-Cherkess Republic, 26—Republic of Karelia, 27—Kemerovo Oblast, 28—Khabarovsk Krai, 29—Republic of Khakassia, 30—Khanty-Mansiy Autonomous Okrug, 31—Kirov Oblast, 32—Komi Republic, 33—Kostroma Oblast, 34—Krasnodar Krai, 35—Krasnoyarsk Krai, 36—Kurgan Oblast, 37—Kursk Oblast, 38—Leningrad Oblast, 39—Lipetsk Oblast, 40—Magadan Oblast, 41—Mariy-El Republic, 42—Republic of Mordovia, 43—City of Moscow, 44—Moscow Oblast, 45—Murmansk Oblast, 46—Nenets Autonomous Okrug, 47—Nizhegorod Oblast, 48—Republic of North Ossetia-Alania, 49—Novgorod Oblast, 50—Novosibirsk Oblast, 51—Omsk Oblast, 52—Orlov Oblast, 53—Orenburg Oblast, 54—Penza Oblast, 55—Perm Krai, 56—Primorsky Krai, 57—Pskov Oblast, 58—Rostov Oblast, 59—Ryazan Oblast, 60—Sakhalin Oblast, 61—Sakha Republic, 62—Samara Oblast, 63—Saratov Oblast, 64—Smolensk Oblast, 65—Stavropol Krai, 66—Sverdlovsk Oblast, 67—Tambov Oblast, 68—Republic of Tatarstan, 69—Tomsk Oblast, 70—Tula Oblast, 71—Tyva Republic, 72—Tver Oblast, 73—Tyumen Oblast, 74—Republic of Udmurtia, 75—Ulyanovsk Oblast, 76—Vladimir Oblast, 77—Volgograd Oblast, 78—Vologda Oblast, 79—Voronezh Oblast, 80—Yamalo-Nenets Autonomous Okrug, 81—Yaroslavl Oblast, 82—Jewish Autonomous Oblast, 83—Zabaykalsky Krai.

**Fig 2:**
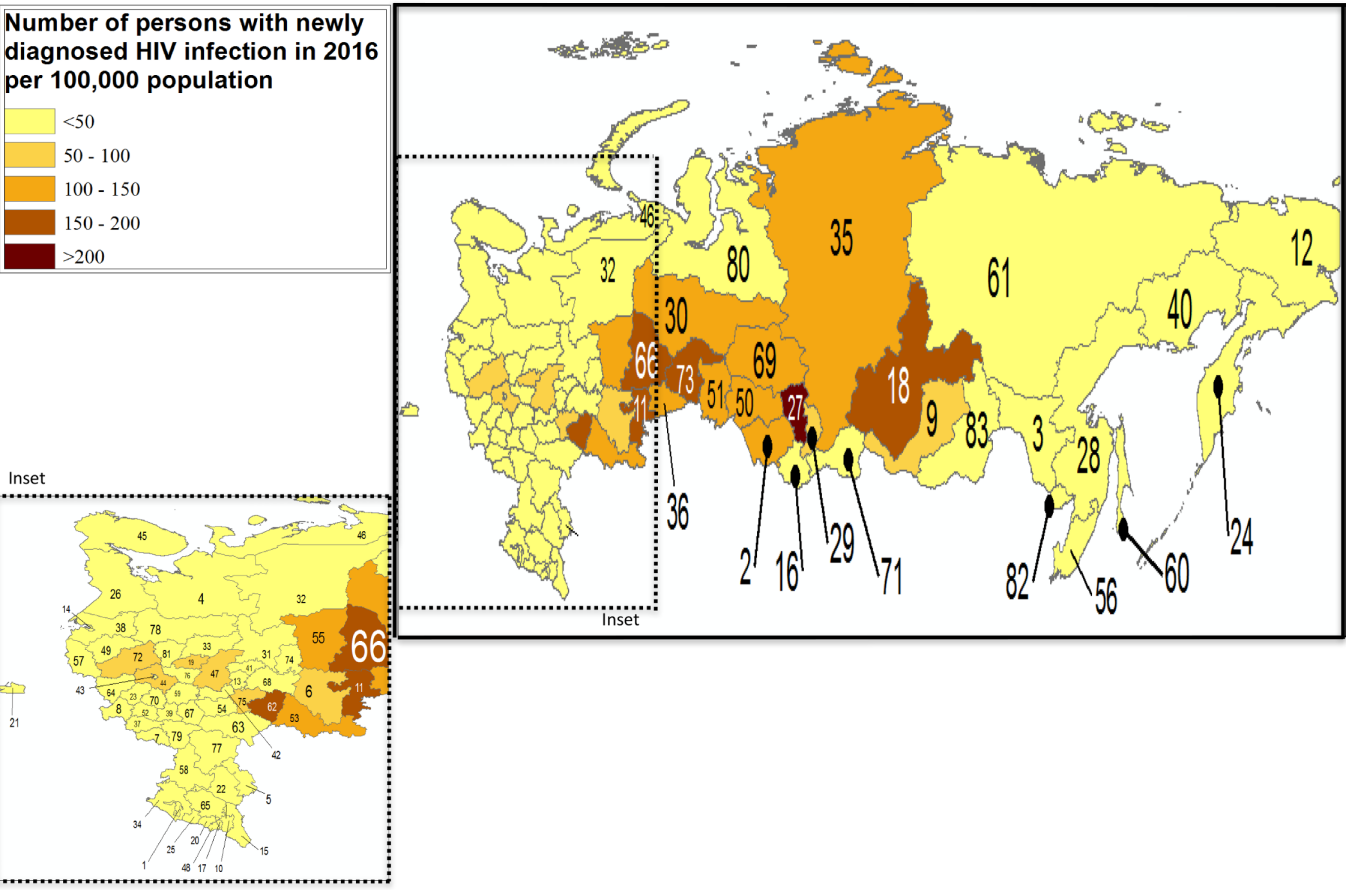
Annual new HIV diagnoses reported in 2016 per 100,000 population [2,6]. Labels: 1—Republic of Adygea, 2—Altai Krai, 3—Amur Oblast, 4—Arkhangelsk Oblast, 5—Astrakhan Oblast, 6—Republic of Bashkortostan, 7—Belgorod Oblast, 8—Bryansk Oblast, 9—Republic of Buryatia, 10—Republic of Chechnya, 11—Chelyabinsk Oblast, 12—Chukotka Autonomous Okrug, 13—Chuvash Republic, 14—City of St. Petersburg, 15—Republic of Dagestan, 16—Altai Republic, 17—Republic of Ingushetia, 18—Irkutsk Oblast, 19—Ivanovo Oblast, 20—Kabardino-Balkar Republic, 21—Kaliningrad Oblast, 22—Republic of Kalmykia, 23—Kaluga Oblast, 24—Kamchatka Krai, 25—Karachay-Cherkess Republic, 26—Republic of Karelia, 27—Kemerovo Oblast, 28—Khabarovsk Krai, 29—Republic of Khakassia, 30—Khanty-Mansiy Autonomous Okrug, 31—Kirov Oblast, 32—Komi Republic, 33—Kostroma Oblast, 34—Krasnodar Krai, 35—Krasnoyarsk Krai, 36—Kurgan Oblast, 37—Kursk Oblast, 38—Leningrad Oblast, 39—Lipetsk Oblast, 40—Magadan Oblast, 41—Mariy-El Republic, 42—Republic of Mordovia, 43—City of Moscow, 44—Moscow Oblast, 45—Murmansk Oblast, 46—Nenets Autonomous Okrug, 47—Nizhegorod Oblast, 48—Republic of North Ossetia-Alania, 49—Novgorod Oblast, 50—Novosibirsk Oblast, 51—Omsk Oblast, 52—Orlov Oblast, 53—Orenburg Oblast, 54—Penza Oblast, 55—Perm Krai, 56—Primorsky Krai, 57—Pskov Oblast, 58—Rostov Oblast, 59—Ryazan Oblast, 60—Sakhalin Oblast, 61—Sakha Republic, 62—Samara Oblast, 63—Saratov Oblast, 64—Smolensk Oblast, 65—Stavropol Krai, 66—Sverdlovsk Oblast, 67—Tambov Oblast, 68—Republic of Tatarstan, 69—Tomsk Oblast, 70—Tula Oblast, 71—Tyva Republic, 72—Tver Oblast, 73—Tyumen Oblast, 74—Republic of Udmurtia, 75—Ulyanovsk Oblast, 76—Vladimir Oblast, 77—Volgograd Oblast, 78—Vologda Oblast, 79—Voronezh Oblast, 80—Yamalo-Nenets Autonomous Okrug, 81—Yaroslavl Oblast, 82—Jewish Autonomous Oblast, 83—Zabaykalsky Krai.

Cumulative Russian federal data through mid-2017 are available on Russian language websites from several oblasts, a Federal AIDS Center report, and from *Rosstat*, the Russian Federal Statistics Bureau [2,5–7]. Russian federal data are reported as cumulative diagnoses and as new diagnoses per 100,000 population. We used these data to generate burden maps of reported prevalence and of new diagnoses, by oblast, across the RF (Fig 1 and Fig 2). Neither measure can be said to yield true HIV prevalence or incidence infection estimates, since they do not account for deaths (in the case of cumulative infections) or estimate the rate of new infections, including among those people never tested. Nevertheless, these available data do allow interpretation of the regional disparities and trends in Russia’s HIV epidemic.

### The current epidemiology of HIV-1

In Fig 1, cumulative HIV diagnoses in 2016 are shown per 100,000 population across the RF’s subjects. Fig 2 shows annual new HIV diagnoses reported in 2016 per 100,000 population across the same territory. There is marked geographic variation in the HIV burden across the country, ranging from a high of 228.8 new diagnoses/100,000 in the Kemerovo Oblast in Southwestern Siberia to less than 5/100,000 in the southern province of Kalmykia, as well as in the Republic of Tyva bordering Mongolia. Among the 10 provinces with the greatest HIV burden in terms of new diagnoses is a geographically large cluster of 6 federal subjects in Eastern and Western Siberia (“federal subjects” is the official term referring to republics, oblasts, and cities of Federal significance in the RF). These include several provinces with contiguous borders, including Kemerovo and its neighbors Irkutsk, second most affected with 163.6/100,000 population; Tyumen, with 150.5/100,000 infections; Tomsk, with 138/100,000; Novosibirsk, with 137.1/100,000; and Krasnoyarsk with 129.5/100,000. This expansive region of 6 provinces sits north of Kazakhstan and on one of the principal overland heroin and opioid trafficking routes out of Afghanistan, the world’s largest producer of illicit opioids in 2016 [8]. Afghanistan lies to the south of the 6 federal subjects in the Siberian cluster. A second heroin and opioid trafficking route out of Afghanistan also supplies illicit opiates to the RF, this a route through the Balkans and into Southwestern Russia [8].

The Siberian region also harbors the highest rates of tuberculosis (TB) infection, with incidence estimates that are 1.6 times higher than those of the rest of the country [9]. Multidrug-resistant tuberculosis (MDR-TB) is also a significant public health threat in this region and in the RF more widely. People who inject drugs have been found to be the group at highest risk for acquisition of TB and MDR-TB in the RF, Belarus, and Ukraine [10].

The most common risk for HIV infection across the country and in the Siberian region is exposure through sharing of injecting equipment among people who inject drugs (PWID) [1,11,12]. In the 2016 Federal AIDS Center surveillance, PWID accounted for the largest proportion of new diagnoses of any risk group at 48.8%; followed by heterosexual sex at 48.7%; homosexual sex, reportedly 1.5%; and 0.8% among perinatally infected infants [2]. These proportions are of uncertain validity, however, and confidence intervals were not reported. Adding to these uncertainties, the proportion of infections due to “undetermined diagnoses” in the data reported by Federal Scientific for the Prevention and Combat of AIDS is high and has been increasing for the past 10 years [13]. In 2014, 55.4% of new infections were reported as “no data” with regards to the source of infection [13]. Under-reporting of risks, especially same-sex behavior, given existing laws banning the sharing of information related to homosexuality and official allowance of stigma towards gay men and other men who have sex with men (MSM) [14], is highly likely to affect these results [15,16].

The epidemiology of HIV in the RF after 3 decades of spread is complex and challenging to analyze. HIV burdens among MSM in the RF were reported to be in the 4%–6% range in 2012, yet a large respondent driven sample of some 1,300 MSM in Moscow found a prevalence of 15.6% (11.6% after RDS weighting) and reported that some three-quarters of these men were not previously aware of their HIV status and had not had recent HIV testing [17]. These and other studies have consistently found higher burdens of HIV infection among key populations than what has been reported in the Russian surveillance and larger numbers of key populations. This suggests that many of those most at risk for HIV are not captured in the Russian national data and that these data are likely underestimates of the true burden [18]. In 2014, Pokrovskaya and colleagues from the Russian Federal AIDS Center suggested that only 51% of people living with HIV in the country in 2013 had been diagnosed with their infection [3,19]. If generalizable and valid, this could bring the estimated cumulative number of HIV infections in the RF to more than 2 million.

### Unmet prevention needs

HIV prevention measures have lagged markedly in the RF. This has been particularly true for the large population of PWID, since evidence-based drug treatment for opioid dependence is not available [20,21]. The RF continues to ban any opioid agonist therapy, including prescription use of methadone and buprenorphine, despite these agents being on WHO’s essential drug list [21–23].

The passage of an antigay propaganda law in 2013 further restricted an already difficult environment for gay men and other MSM in Russia, also reducing prevention and treatment access for these men [15–17]. After passage of the 2013 law, it became illegal to post or discuss information for gay men and other MSM, even on informational websites in Russia, further restricting information. Preexposure prophylaxis is not available, and treatment coverage for MSM is remarkably low, even in Moscow, where only some 9% of MSM living with HIV infection were found to be on antiretroviral therapy in 2014 [17].

## Conclusions

The RF is undergoing a severe, widespread, and geographically dispersed HIV epidemic. There is a very large 6-region cluster in Eastern and Western Siberia that is now the most affected part of this vast country. HIV prevalence and incidence are difficult to directly deduce from the available reporting data, but there is enough evidence to suggest that Russia’s epidemic is uncontrolled and worsening in 2017. It is disturbing that deaths are rising rapidly in an upper middle-income country that could, and should, be doing much better in the provision of prevention, treatment, care, and support for its citizens, especially those at high risk of HIV infection [24].

The continuous growth of the Russian HIV epidemic is a failure of public policy and practice. The current list of interventions with demonstrable efficacy in reducing HIV spread and improving treatment outcomes includes opioid agonist substitution therapy, needle and syringe exchanges, treatment as prevention, preexposure prophylaxis, and tailored interventions for key populations including PWID, MSM, sex workers, prisoners, and migrants. In the RF, all of these interventions are either not available or are unavailable at the scale necessary to control HIV. This is a true public health crisis and one that could largely have been avoided. Unless evidence-based prevention measures aimed at the most at-risk population groups are brought to scale in the RF, and unless access to treatment is significantly increased for all HIV-infected people, the likelihood of greater HIV incidence, and consequently greater AIDS morbidity and mortality, will only increase.

## Abbreviations

ECDC: European Centre for Disease Prevention and Control
MDR-TB: multidrug-resistant tuberculosis
MSM: men who have sex with men
PWID: people who inject drugs
RF: Russian Federation
TB: tuberculosis
